# Case Report: Nephrotic syndrome with minimal change disease during anti-tuberculosis treatment and tiopronin use

**DOI:** 10.3389/fmed.2026.1807135

**Published:** 2026-08-10

**Authors:** Heng Liao, Fei Yan, Xingxia Liu

**Affiliations:** 1 Department of Pharmacy, The Fourth People's Hospital of Chengdu, Chengdu, China; 2 Department of Clinical Laboratory, The Clinical Hospital of Chengdu Brain Science Institute, MOE Key Lab for Neuroinformation, University of Electronic Science and Technology of China, Chengdu, China; 3 Department of Clinical Laboratory, The Fourth People's Hospital of Chengdu, Chengdu, China; 4 Department of Pharmacy, Sichuan Provincial People’s Hospital, Chengdu, China

**Keywords:** anti-tuberculosis therapy, drug withdrawal, drug-associated kidney disease, minimal change disease, nephrotic syndrome, tacrolimus therapeutic drug monitoring, tiopronin

## Abstract

**Background:**

Hepatoprotective agents are often added during prolonged anti-tuberculosis (anti-TB) therapy, and medication-associated nephrotic syndrome may be under-recognized.

**Case:**

A 31-year-old man with pulmonary tuberculosis received long-term anti-TB therapy (rifapentine, isoniazid, and ethambutol) and continuous tiopronin (0.2 g three times daily). He developed edema, foamy urine, and oliguria. Pre-admission tests showed proteinuria (3+), serum albumin 19.6 g/L, and serum creatinine 161 μmol/L. On admission, urine protein was 4+, serum albumin 20.6 g/L, serum creatinine 142.9 μmol/L, and estimated glomerular filtration rate (eGFR) 58.86 mL/min, with hyperlipidemia. Kidney biopsy confirmed minimal change disease (MCD).

**Interventions and outcome:**

All anti-TB drugs and tiopronin were discontinued, and tacrolimus was initiated after biopsy. Anti-TB therapy was reintroduced as isoniazid monotherapy. Renal function improved rapidly (serum creatinine 55 μmol/L; eGFR 128.1 mL/min at discharge), and dipstick proteinuria became negative on short-term follow-up. The tacrolimus trough concentration was 3.6 ng/mL.

**Conclusion:**

The improvement after withdrawal of multiple suspected agents and continued recovery after reintroduction of isoniazid alone support a drug-associated etiology, but do not allow definitive attribution to a single causative drug. Routine renal monitoring should accompany hepatoprotective add-on therapy during anti-TB treatment.

## 1 Introduction

Long-course anti-tuberculosis (anti-TB) therapy is typically delivered as combination regimens and often must be maintained for months. In China, drug-susceptible pulmonary TB is generally treated with a 2-month intensive phase of isoniazid, rifampicin, pyrazinamide, and ethambutol, followed by a 4-month continuation phase with isoniazid and rifampicin (2HRZE/4HR) (1). The regimen and duration may be extended or modified in selected situations (e.g., disseminated disease or post-surgical management) and when adverse events limit tolerance. In routine practice, hepatoprotective agents are frequently co-prescribed to address fluctuations in liver function and to help patients remain on treatment. Clinicians may reasonably expect such supportive therapy to improve tolerability without introducing additional clinically meaningful harm, because uninterrupted anti-TB treatment is central to disease control and to preventing relapse or resistance. Adverse events that force treatment interruption can compromise outcomes, while unrecognized drug toxicity can lead to avoidable organ injury.

Despite careful liver monitoring, renal safety in this setting receives less consistent attention. When edema, proteinuria, or rising creatinine occurs during anti-TB treatment, the presentation may be attributed to infection-related stress, nutritional issues, or coincidental primary kidney disease— particularly in patients with complex medication exposure. In addition, “hepatoprotective” add-on agents are sometimes perceived as low-risk supportive drugs and may be underweighted during causality assessment.

Medication-triggered podocytopathies, including minimal change disease (MCD), can present as nephrotic syndrome and may be reversible if the offending exposure is withdrawn early (2, 3). However, under real-world polypharmacy, identifying the culprit agent is difficult, and case de-scriptions may lack a reproducible chain of evidence linking exposure, biopsy phenotype, and re- sponse to withdrawal and re-exposure. Clinicians therefore need a practical approach to recognize suspected drug-associated nephrotic syndrome promptly and to reconstruct anti-TB therapy safely after stabilization.

Here, we present a biopsy-confirmed MCD case manifesting as nephrotic syndrome with renal dysfunction during prolonged anti-TB therapy with tiopronin exposure. We aim to document the exposure–biopsy–outcome trajectory, clarify the role of simultaneous withdrawal of multiple suspected medications and subsequent isoniazid reintroduction in clinical interpretation, and propose an actionable renal monitoring pathway when hepatoprotective add-on therapy is used during anti-TB treatment.

## 2 Case presentation and clinical course

### 2.1 Case presentation

A 31-year-old man (height 174 cm, weight 68 kg) with pulmonary tuberculosis had received anti-TB therapy since March 2019. He initially completed a prolonged 3HRZE/9HRE regimen for pulmonary TB as recorded in the medical chart. In April 2020, he was diagnosed with tuberculous empyema and underwent thoracic surgery, including right upper lobectomy and wedge resection. Anti-TB therapy was continued postoperatively and modified because of intolerance, including hepatotoxicity. Since April 2020, tiopronin 0.2 g three times daily was added as hepatoprotective therapy because of fluctuating liver function and was continued thereafter. From March 2021, his outpatient maintenance regimen consisted of rifapentine 0.6 g twice weekly, isoniazid 0.3 g once daily, and ethambutol 0.75 g once daily, with continued tiopronin 0.2 g three times daily.

**Figure 1:**
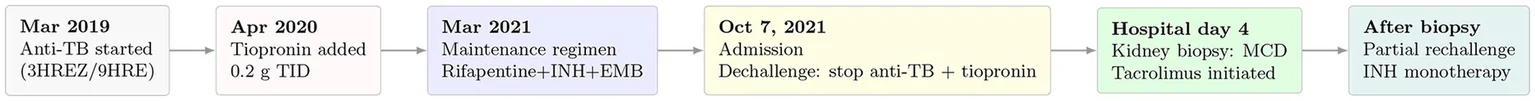
Medication exposure timeline and key clinical events.

One week before admission, he developed progressive bilateral lower-limb edema, morning facial edema, foamy urine, and oliguria (approximately 400–500 mL/day), accompanied by nausea, vom- iting, and abdominal distension. Pre-admission testing on 3 October 2021 showed proteinuria (3+), serum albumin 19.6 g/L, serum creatinine 161 μmol/L, and eGFR 46.4 mL/min. As symptoms did not improve after intravenous therapy, he was admitted on 7 October 2021 with nephrotic syndrome and renal dysfunction. Tiopronin had been continued as prophylactic hepatoprotection despite normalization of liver enzymes at admission (see Figure 1). Serial blood pressure measurements during hospitalization showed systolic values of 118–131 mmHg and diastolic values of 60–85 mmHg, without sustained hypertension.

Timeline of anti-tuberculosis regimens and tiopronin exposure, aligned with admission, simultaneous withdrawal of anti-tuberculosis agents and tiopronin, kidney biopsy confirming minimal change disease, tacrolimus initiation, and reintroduction of isoniazid alone as maintenance therapy. The timeline shows temporal associations but does not establish a single causative drug because multiple agents were withdrawn simultaneously.

### 2.2 Diagnostic assessment

Admission laboratory findings supported a nephrotic syndrome phenotype with impaired renal function: urine protein 4+, serum albumin 20.6 g/L, serum creatinine 142.9 μmol/L, eGFR 58.86 mL/min, urea 12.99 mmol/L, and uric acid 521 μmol/L, accompanied by secondary hyperlipidemia (total cholesterol 6.75 mmol/L; LDL-C 4.19 mmol/L). Quantitative proteinuria assessment confirmed nephrotic-range proteinuria, with a 24-h urinary total protein of 6.044 g/24 h on Oct 11, 2021. Liver enzymes were within normal ranges (AST 19 U/L; ALT 14 U/L). Complement levels were not decreased (C3 1.34 g/L; C4 0.323 g/L). Autoimmune screening showed positive ANA and negative MPO-ANCA. Although ANA was positive, complement levels were not decreased, MPO-ANCA was negative, and kidney biopsy did not show features supporting immune-complex glomerulonephritis. Viral serologies for HBV, HCV, and HIV were negative. A kidney biopsy during hospitalization demonstrated histopathology consistent with MCD. eGFR was calculated using the CKD-EPI equation adapted for Chinese patients. Because serum creatinine changed during the acute phase, eGFR was interpreted cautiously, and serial serum creatinine trends were used as the primary marker of renal recovery.

### 2.3 Therapeutic intervention

On admission, all anti-TB agents and tiopronin were withdrawn simultaneously, and the patient received a low-salt diet, diuretics, and supportive care. Given mildly elevated inflammatory markers (ESR 56 mm/h, CRP 5.85 mg/L, and PCT 0.22 ng/mL) and gastrointestinal symptoms, ceftizoxime sodium was administered for a short course.

After biopsy confirmation of MCD, corticosteroids were not used because of the patient’s tuberculosis history and the concern that systemic corticosteroid exposure could increase the risk of infection reactivation or dissemination. Tacrolimus was therefore selected as a steroid-sparing option to control proteinuria, with therapeutic drug monitoring planned to improve safety. Anti-TB treatment was cautiously reconstructed by resuming oral isoniazid alone as maintenance therapy. Diuretic therapy was adjusted according to clinical response (torasemide combined with hydrochlorothiazide and spironolactone).

### 2.4 Follow-up and outcomes

Renal function improved rapidly after treatment initiation. Serum creatinine decreased from 142.9 μmol/L at admission to 130.4 μmol/L on hospital day 5 and 73.8 μmol/L on day 8, reaching 55 μmol/L before discharge; eGFR increased accordingly from 58.86 mL/min to 62.4, 117, and 128.1 mL/min. Proteinuria improved in parallel (urine protein from 4 + to 3+). Serum albumin recovered more slowly (20.6 g/L at admission, 17.2 g/L on day 5, 16.0 g/L on day 8, and 17.9 g/L before discharge). He was discharged on Oct 18, 2021.

**Figure 2:**
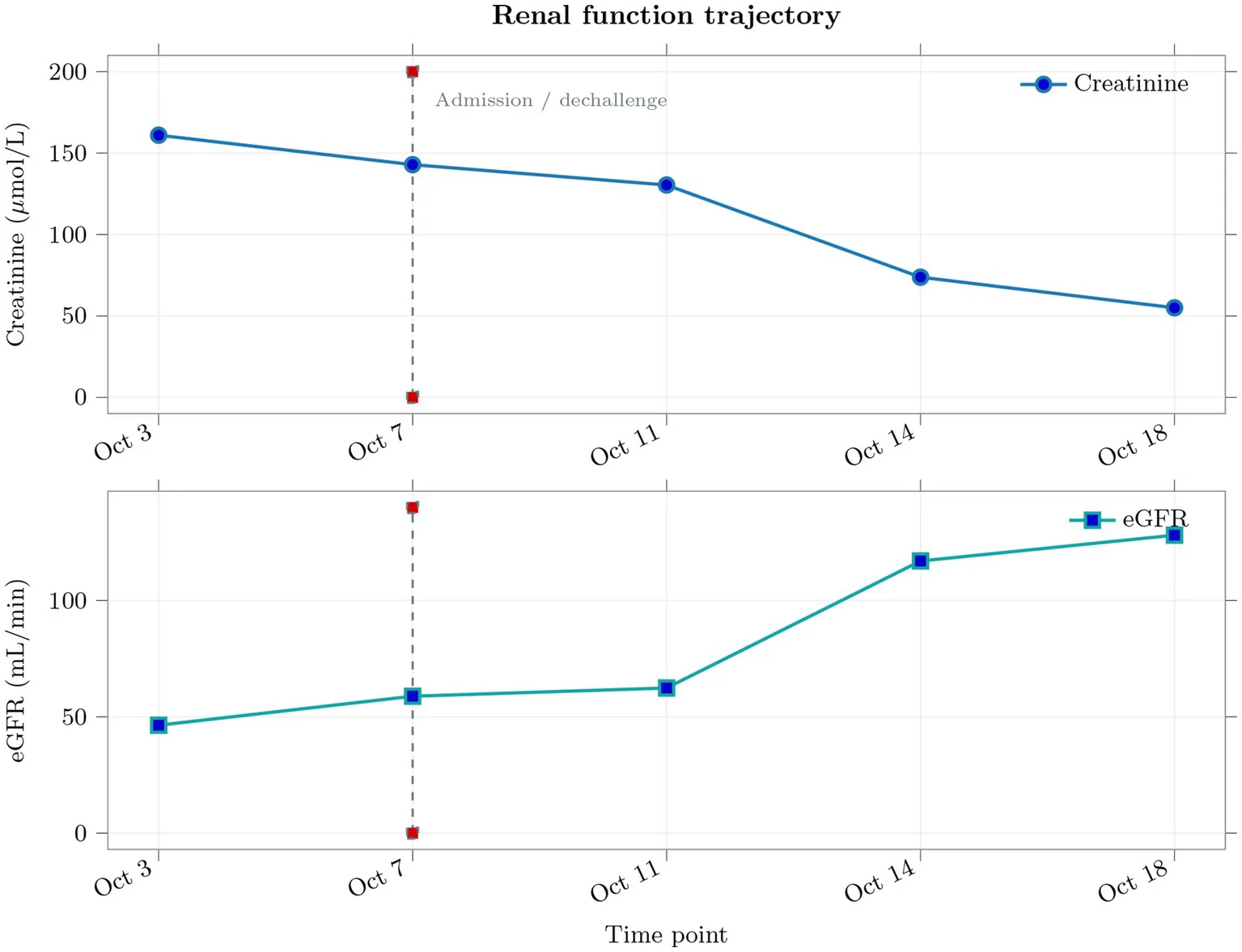
Renal function trajectory before and after drug withdrawal (dechallenge). Serum creatinine and estimated glomerular filtration rate (eGFR) over time. The vertical dashed line indicates admission and implementation of dechallenge (discontinuation of anti-tuberculosis agents and tiopronin).

**Figure 3:**
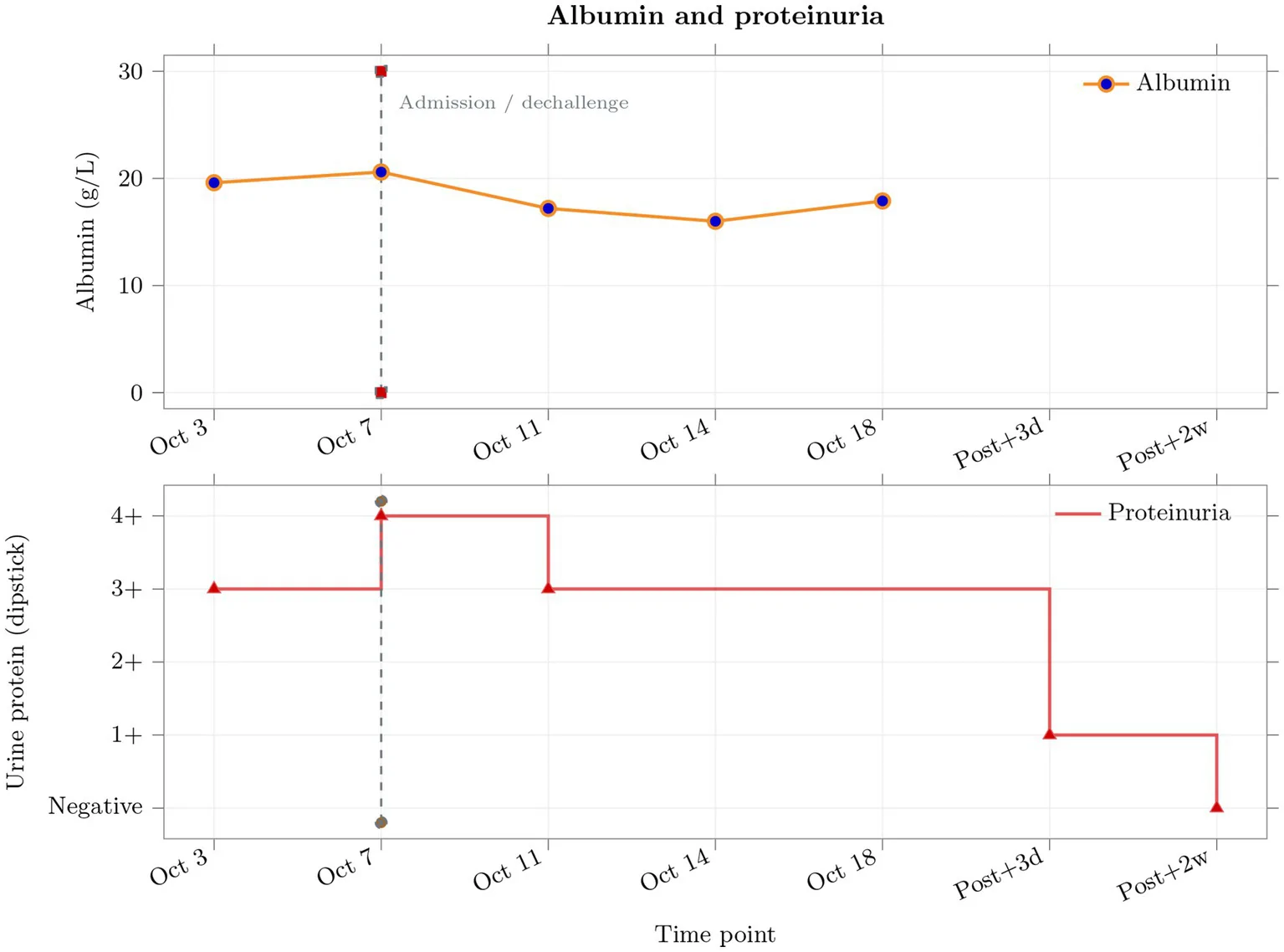
Longitudinal changes in serum albumin and dipstick proteinuria. Serum albumin and urine dipstick proteinuria over time from pre-admission to post-discharge follow-up. Proteinuria improved earlier than serum albumin recovery, consistent with a delayed normalization of hypoalbuminemia in nephrotic syndrome.

Short-term post-discharge follow-up showed continued improvement: urine protein decreased to 1 + on day 3 post-discharge and became negative at subsequent outpatient review. Because post-discharge proteinuria was assessed by urine dipstick, these follow-up results were interpreted as semi-quantitative indicators rather than precise measurements of protein excretion. The tacrolimus trough concentration was 3.6 ng/mL (see Figures 2, 3).

## 3 Discussion

### 3.1 Drug-associated MCD: interpretation under polypharmacy

This case is best framed as drug-associated MCD during prolonged anti-TB therapy with tiopronin exposure, rather than toxicity from a proven single agent. The improvement in renal function and proteinuria after simultaneous withdrawal of anti-TB agents and tiopronin supports a medication-related process, but does not identify the culprit drug. Tiopronin is the leading suspected agent because prior reports have linked it to nephrotic syndrome and biopsy-proven MCD (4). Rifapentine cannot be excluded, although evidence for rifapentine-associated MCD is much more limited than for rifampicin (5, 6). Continued recovery after isoniazid reintroduction suggests that isoniazid was less likely to be the main driver, but this was not a true rechallenge of tiopronin or rifapentine.

### 3.2 Therapeutic strategy and drug–drug interaction considerations

Adult MCD is traditionally treated with high-dose glucocorticoids as first-line therapy; however, individualized risk–benefit assessment is required, particularly in patients with a tuberculosis history (2). In this patient, corticosteroids were avoided because systemic steroid exposure could increase the risk of infection reactivation or dissemination. A tacrolimus-based, steroid-sparing strategy with therapeutic drug monitoring was therefore selected (7). Notably, the tacrolimus trough concentration during follow-up was 3.6 ng/mL, below the typical target range used for many glomerular diseases. The continued clinical improvement despite this relatively low exposure suggests that withdrawal of the suspected medications may have been the major driver of recovery, while tacrolimus may have served as adjunctive therapy rather than the sole determinant of improvement.

### 3.3 Practice implications: renal pharmacovigilance during hepatoprotective add-on therapy

A practical takeaway is that hepatoprotective add-on therapy should be treated as a meaning- ful exposure in pharmacovigilance rather than as a benign accessory. During long-term anti-TB therapy, renal monitoring should run in parallel with liver monitoring. A pragmatic pathway may include: (i) routine surveillance of urinalysis/proteinuria, serum albumin, serum creatinine/eGFR, and lipid profile; (ii) predefined triggers such as new-onset or progressive proteinuria, declining albumin, rising creatinine, or falling eGFR; and (iii) prompt medication reconciliation, withdrawal of suspected agents, and kidney biopsy when severity or clinical course warrants (2).

### 3.4 Limitations

This case has several limitations. First, because all anti-TB agents and tiopronin were withdrawn simultaneously, the clinical course cannot identify a single causative drug. Second, although nephrotic-range proteinuria was confirmed by 24-h urinary protein measurement during hospitalization, some follow-up assessments relied on urine dipstick testing, which is semi-quantitative and less precise than UPCR, UACR, or repeated 24-h urine collection. Third, although complement levels, ANCA testing, viral serologies, and kidney biopsy were reviewed, the autoimmune evaluation was not fully comprehensive; therefore, secondary causes of podocytopathy cannot be excluded with absolute certainty. Finally, follow-up was short, and long-term relapse risk after treatment reconstruction remains uncertain.

## 4 Conclusion

This case describes nephrotic syndrome with biopsy-proven MCD following prolonged combined anti-TB therapy with tiopronin exposure. Clinical improvement after simultaneous withdrawal of multiple suspected agents supports a drug-associated etiology, but the evidence does not allow definitive attribution to a single causative drug because neither tiopronin nor rifapentine was reintroduced. In clinical practice, hepatoprotective agents should be included in pharmacovigilance and renal safety monitoring alongside core anti-TB regimens.

## Data Availability

The original contributions presented in the study are included in the article/Supplementary material, further inquiries can be directed to the corresponding authors.
